# A social perspective on resilience: social support and dyadic coping in teacher training

**DOI:** 10.1186/s40461-021-00126-y

**Published:** 2021-12-20

**Authors:** Tobias Kärner, Julia Katharina Weiß, Karin Heinrichs

**Affiliations:** 1 Chair of Economic and Business Education (560A), University of Hohenheim, Fruwirthstraße 47, 70593 Stuttgart, Germany; 2 University of Education Upper Austria, Kaplanhofstr. 40, 4020 Linz, Austria

**Keywords:** Teacher training, Social support, Dyadic coping, Relational resilience, Coping strategies

## Abstract

Stress in teaching and teacher training is a well-known issue and stress management during teacher training may not only be affected by individual coping efforts, but also determined by private and work-related networks the individual is integrated in. In that regard, our article aims firstly to identify sources of social support in the German teacher training system and secondly to analyze interdependencies in dyadic coping interactions based on the Actor-Partner Interdependence Model. On the basis of questionnaire data from 307 German trainees and qualified teachers from vocational and general schools, we found that mentors, partners, fellow trainees, colleagues at school, parents, and good friends were named as the most supportive reference persons during teacher training. In a follow-up survey, data from 49 sources of support were obtained, which could be assigned to the corresponding (trainee) teachers (in the sense of support recipients). These dyads thus form the basis for the analysis of dyadic coping interdependencies. The results of the moderator analyses show, among other things, that support recipients who prefer the coping strategy palliative emotion regulation tend to react rather sensitively to contrary coping strategies of the source of support with regard to their stress symptoms. Social interactions in this respect can represent both protective as well as risk factors. Therefore, a system of complex social interdependencies must be considered when analyzing relational resilience among prospective teachers.

## Introduction

Physical and mental health problems caused by *stress* in the teaching profession and in teacher training are well-known issues in the educational system (e.g., Chaplain 2008; Gardner 2010; Harmsen et al. 2018). In that regard, teacher stress is defined “as the experience by a teacher of unpleasant emotions, such as tension, frustration, anxiety, anger and depression, resulting from aspects of his work as a teacher” (Kyriacou 1987, p. 146). Being a teacher is demanding and confronts some teachers with challenges that exceed their own coping resources and result, for example, in psychosomatic symptoms (Capel 1992; Kieschke and Schaarschmidt 2008). Contextual resources along with the use of certain strategies have been found to contribute to resilience outcomes in teacher training (Mansfield et al. 2016). With respect to that, the concept of resilience refers “to a dynamic process encompassing positive adaptation within the context of significant adversity” (Luthar et al. 2000, p. 543). In order to effectively cope with adversities, coping or stress management hereby generally refers to the process of dealing with stressful situations, meaning that individuals perform purposeful and intentional actions to meet and overcome stressful demands (Folkman and Moskowitz 2004; Kyriacou and Sutcliffe 1978; Lazarus 1966; Lazarus and Folkman 1984; Schwarzer and Schwarzer 1996). The *individual* way of dealing with stressors and the applied coping strategies can serve as an explanatory approach for individually varying strain in teacher training (Kärner et al. 2021a; Braun et al. 2020; Lindqvist 2019).

However, an individual perspective of resilience and coping with stress falls short, as stress and coping processes must be considered within a system of *social interdependencies*, which in turn provide the context of individual experience and individual coping efforts (e.g., Boldrini et al. 2019; Buchwald 2002; Sappa et al. 2019; Schumacher 2002; Sembill and Kärner 2018). Johnson et al. (2014) also suggest that, with the aim of exploring early career teacher resilience and promoting beneficial outcomes, one must acknowledge dynamic interactions and take social contexts into account.

Thus, apart from individual coping efforts, *social support* is considered to be of central importance when it comes to coping with stress. In general, social support has proven, for example, to have a positive effect on psychological well-being and health (e.g., Benight and Bandura 2004; House et al. 1988; Kassis et al. 2019). In the teaching context, Kärner et al. (2016) found in a diary study that seeking social support was the most mentioned coping strategy of teachers in handling daily work-related demands. Furthermore, existing studies indicate that social support can reduce stress in teacher training (e.g., Richter et al. 2011; Warwas et al. 2016). In order to investigate the impact of social support on stress perception, it seems necessary to broaden the focus beyond the perspective of an individual (Bodenmann 1997; Knoll and Schwarzer 2005; O’Brien and DeLongis 1997). Resilience in that regard encompasses “the dynamic process whereby characteristics of individual teachers and of their personal and professional contexts interact” (Mansfield et al. 2016, p. 7–8). When considering dyads consisting of a *support recipient* and a *source of support*, interdependencies in different support constellations can be analyzed. Here, it is of interest to explore the extent to which a support provider and his or her actions affect the stress experience of his or her dyadic interaction partner. The reciprocal interplay of stress and coping processes between two interaction partners is referred to as *dyadic coping* (cf. Bodenmann 1997; Cook and Kenny 2005; Kenny and Cook 1999). In addition, and in order to examine the role of coping strategies in the experience of stress, the effects of specific coping efforts of both persons involved in the dyad are of interest.

Concerning effects of dyadic coping on stress and well-being, Falconier and Kuhn (2019, p. 19) reviewed a total of 139 studies on dyadic coping in couples; the authors concluded that positive forms of coping are “beneficial for each partner’s individual and relational well-being when they cope with stress in general.” Fuenfhausen and Cashwell (2013) found that dyadic coping is significantly negatively correlated with perceived stress in counseling graduate students. However, the authors did not differentiate between different kinds of coping strategies. Buchwald and Schwarzer (2003) examined dyadic coping in student–teacher interactions; the authors found that dyadic coping acts as a protective factor in oral exam situations. In reviewing the existing literature, it may be cautiously stated that the current approach is novel, as support dyads have not, to our knowledge, been considered in previous research on teacher education.

**Fig. 1:**
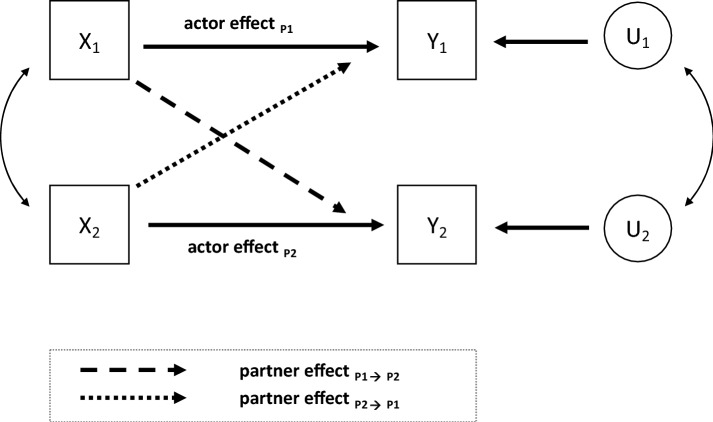
Actor-Partner Interdependence Model (APIM). X_1/2_ = independent variable, Y_1/2_ = dependent variable; U_1/2_ = residual; Own illustration based on Cook and Kenny (2005, p. 102)

**Fig. 2:**
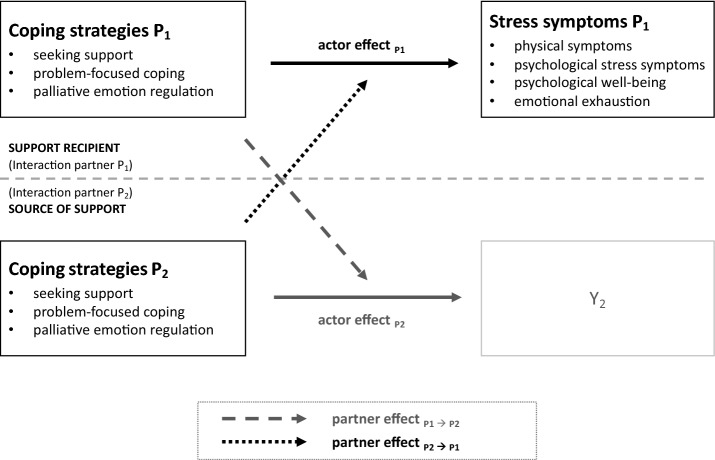
Summarizing analysis model. Grayed out elements are not examined in this study, these are only used to provide a complete representation of the modified APIM; Own illustration based on Cook and Kenny (2005, p. 102)

Against this background, we aim to identify sources of social support in teacher training. Furthermore, the interplay of the coping strategies of the support recipient and the source of support are examined in relation to stress symptoms reported by the support recipient. For this purpose, the concept of dyadic support is applied. To pursue this research aim, first of all, theoretical and empirical findings of social support and dyadic interactions are described in view of the Actor-Partner Interdependence Model (APIM) (Cook and Kenny 2005; see Fig. 1 in “General modeling of dyadic interactions“ Section). Then, potential sources of support and specific coping strategies are discussed. Eventually, dyadic support and individual stress management can be unified in the adapted APIM, i.e., the summarizing analysis model of this study (see Fig. 2 in “Summarizing analysis model” Section). The results of the empirical analysis form the basis for the final discussion of findings. Lastly, implications for further research and for practice are pointed out.

## Social support and dyadic coping as resources in teacher training

Overall, we refer back to two central modeling issues. On the one hand, we are interested in social support during teacher training and how it affects dyadic support interactions. On the other hand, and within this notion of dyadic support, we focus on specific coping strategies and thus individual coping efforts. Originally, Bodenmann (2000) conceptualized dyadic coping as social support within a committed partnership (Gmelch and Bodenmann 2007). This paper does not exclusively refer to the “significant other” as in the partner or spouse, but extends this concept to a person in teacher training who is perceived as particularly supportive, since it has been found that relationships both within and outside of the professional context constitute important contextual resources enhancing teacher resilience (Mansfield et al. 2016). Before elaborating on these components in more detail, the first part of this section presents the context of German teacher education.

### The context of German teacher training

In Germany, the structure of teacher education is organized into two basic parts. The initial phase takes place at a university or at a college of education. The preparatory service represents the second, practical phase of teacher education, also referred to as teacher training. Overall, the duration of this preparatory service in Germany varies between 12 and 24 months (Kärner et al. 2019; Klusmann et al. 2012). The legal regulation of education is subject to the cultural sovereignty of the individual federal states, which means that the length of the teacher training as well as the content, the goals, and the organizational structure vary from state to state (Terhart 2000). Yet, commonalities can be identified nationwide. Typically, this second phase of teacher education, which we focus on, takes place at two learning venues, the college of didactics and teacher education, and the training school (Bellenberg and Thierack 2003). Thus, similar to apprenticeship training in Germany, teacher training follows the principle of duality and offers the possibility of “learning on the job” (Halász et al. 2004, p. 32). At the training school, trainee teachers are supervised by a specific teacher (a mentor or supervising teacher), in addition to the principal and the teaching staff in general. At the college of didactics and teacher education, seminar teachers are responsible for training in didactics and teaching competence in specific subjects (Bellenberg and Thierack 2003).

### General modeling of dyadic interactions

Social interactions represent a central component of teacher resilience. Therefore, the relational concept of resilience is understood to as “a dynamic process within a social system of interrelationships influenced by the interaction between the individual and the environment” (Gu 2014, p. 507). Supportive interactions provide an important contextual resource in teacher training, facilitating the coping efforts of trainee teachers (Richter et al. 2011; Warwas et al. 2016). In general, social support encompasses the interaction between two or more people aimed at resolving or alleviating a problem state that creates distress in an individual (Knoll and Schwarzer 2005). Since these interpersonal interactions involve not only the trainee but both the support recipient *and* the source of support, the isolated consideration of the support recipient, i.e., the trainee, provides significantly less information about the effects of the supportive actions than when taking both perspectives into account (Knoll and Schwarzer 2005).

Against this background, we refer to the *Actor-Partner Interdependence Model* (APIM; Fig. 1) for the analysis of social support through dyadic coping.

The APIM is based on the idea that, in dyadic interactions, not only one’s own personality traits predict the behavior of that person, but those of the interaction partner also serve as an explanatory approach for corresponding actions (Cook and Kenny 2005; Kashy and Kenny 2000; Kenny and Cook 1999). Interdependence within such relationships occurs precisely when the behavior, emotions, as well as cognitions of one person affect the behavior, emotions, and cognitions of another individual (Baker and Berenbaum 2011; Kelley et al. 2003; Klauer and Schwarzer 2001). The central components of the APIM are the *actor and partner effects*. The former describes the extent to which an individual’s behavior (X_1_ and X_2_, respectively) influences the subsequent behavior or outcome (Y_1_ and Y_2_, respectively). The partner effect measures the extent to which one person is influenced by another person. Thus, this effect measures a form of the previously described interaction in terms of the interdependence between two people and is therefore *dyadic* by definition (Cook and Kenny 2005; Kenny and Cook 1999).

### Potential sources of support in teacher training

Previous studies on teacher resilience identified and emphasized the significance of both formal and informal processes of support (Papatraianou and Le Cornu 2014). According to Le Cornu (2013), particularly for beginning teachers strong relationships play an important role in sustaining their resilience. Mansfield et al. (2016) point out that support networks seem to be essential in overcoming challenges and enhancing early career teachers’ resilience. In this sense, context is found to “provide important sources of support for resilience” (Beltman 2021, p. 17).

With regard to seeking advice and support, teacher training in Germany offers a variety of interaction partners potentially serving as sources of support. In order to minimize adjustment problems in practice, especially at the beginning of the preparatory teacher traineeship, there are, for example, mentoring programs that provide support to trainee teachers at school (Richter et al. 2011; Warwas et al. 2016). The importance of mentoring with respect to building resilience is also highlighted by Morettini et al. (2020). Therefore, mentors in particular but also other colleagues, the school principal, fellow trainees, or seminar teachers are potential contacts from whom support can originate. Richter et al. (2011) focus on support from mentors and support from fellow trainees. Both sources of support were perceived as important by the participants in the aforementioned study. Support from mentors is associated with a decline in emotional exhaustion and an increase in trainee teacher’s self-efficacy beliefs. There are different results regarding support provided by fellow trainees; some show positive and some show negative effects. Likewise, support from the private surroundings, i.e., non-work sources of support, should not be neglected (Warwas et al. 2016). Especially one’s spouse or partner (cf., Bodenmann 1997, 2000) as well as family and friends play a central role in reducing stress in general, coping with adversities in teacher training, and enhancing resilience through relational connectedness (cf. Beltman 2021; Beltman et al. 2011; Laireiter 1993; Le Cornu 2013; Papatraianou and Le Cornu 2014). According to Dückers-Klichowski (2005), these private sources of support are particularly reliable during teacher training and are therefore considered essential. Klusmann et al. (2008) showed that support from the private surroundings reduces emotional exhaustion among teachers. Overall, a social network of strong relationships in the professional as well as personal environment of beginning teachers is critical in promoting relational resilience (Gu 2014; Mansfield et al. 2016).

Regardless of whether a source of support stems from the school context or the private background, empathy, sympathy, and similarity play a crucial role in the selection of which person one approaches to seek support (McPherson et al. 2001; Verbrugge 1977). Moreover, situational similarities and commonalities in the sociocultural domain increase the chance that another person will be perceived as empathic. Interview results show that early career teachers’ reciprocal engagement in empathic relationships strengthens their resilience (Le Cornu 2013). In this context, it can be assumed that effective supportive behavior comes primarily from people who are socially similarly situated and have been confronted with similar stressors or have already successfully overcome them (McPherson et al. 2001; Thoits 1986). In terms of teacher training, these findings suggest that people within the professional context, in particular, will be called upon to provide support in coping with stressful demands, as they are/were “going through a similar experience” (Le Cornu 2013, p. 6).

### Coping strategies on the part of the support recipient and their source of support

From a process-focused perspective “resilience lies at the interface of person and context, where individuals use strategies to enable them to overcome challenges” (Beltman 2021, p. 15). These individual coping strategies are conceptually closely related to social support (Schwarzer and Knoll 2007; Thoits 1986). Thoits (1984, 1986) suggests conceptualizing social support as coping assistance, i.e., the active participation of others in the stress-coping efforts of the support recipient. The author further argues that processes of social support are closely linked to coping processes, as individuals apply stress-coping strategies that they use themselves when under stress to other people in stressful situations as assistance. It can be assumed that coping strategies used by sources of support in response to their own stressors also have an influence on the support recipients and their attempts to cope, as these coping efforts “may facilitate, constrict or interfere” with the coping strategies of others (O’Brien and DeLongis 1997, p. 161).

In general, coping strategies are action-related and cognitive efforts to deal with stressors (Lazarus 1966; Schwarzer and Schwarzer 1996). Demonstrating resilience is enabled by the appropriate use of adaptive coping strategies (Mansfield et al. 2016). Also, sharing strategies among fellow trainees was found to be a helpful resource (Le Cornu 2013). Wilkinson (1988, p. 185) states that teachers’ coping strategies are “complex patterns of actions which were palliative or direct‐action, used in sequence or simultaneously to combat stress.” According to Lazarus (1993), there are at least two major functions of coping—problem-focused and emotion-focused—that have to be distinguished.

*Problem-focused coping *involves behaviors with a direct effect on the environment to counteract and accordingly change stressors or, ideally, remove them altogether (Folkman and Lazarus 1988). It is understood as active, often immediate problem confrontation and resolution and has been identified as one of the most frequently mentioned resilience strategies (Mansfield et al. 2016). Problem-focused coping strategies in teachers include, for instance, organizing work, setting limits, and solving problems (Aulén et al. 2021). In contrast, emotion-focused coping, also referred to as cognitive-emotional coping, describes thoughts and actions that are intended to control the undesirable feelings created by the stressor and thus indirectly facilitate the stress-inducing event (Lazarus 1993; Thoits 1986). This may include, for instance, self-control techniques, altering perceptions, blocking out negative issues, or emphasizing positive aspects within the predicament or relativizing it (Aulén et al. 2021; Pogere et al. 2019). *Palliative emotion regulation,* which is a form of emotion-focused coping and another important resilience strategy of teachers (Beltman 2021; Mansfield et al. 2016), refers to an inward emotion-regulating activity (Folkman and Moskowitz 2004). Emotion-focused coping strategies in teachers include, for instance, expressing emotions, seeking rest and relaxation, and self-reflection (Aulén et al. 2021). Concerning teachers’ coping profiles, Aulén et al. (2021) found that stressed teachers mainly used emotion-focused coping strategies. Pogere et al. (2019) found in their study that emotion-focused coping is positively correlated with emotional exhaustion whereas problem-focused coping strategies are negatively associated with emotional exhaustion.

In addition to problem-focused and emotion-based coping, *seeking social support* is another basic coping strategy (Gunther 1994). Help-seeking strategies are also mentioned by Mansfield et al. (2016) as enhancing resilience within the dimension of resilience strategies. The need for support may emerge in stressful situations, which manifests itself in approaching others directly (Folkman and Lazarus 1988; Gunther 1994; Lazarus 1993). This mobilization of support can then generate support provision, whereby support can have both a problem-focused and an emotion-oriented character (Knoll and Schwarzer 2005). Seeking support in terms of social problem-focused coping in teachers includes sharing work, planning and working together, asking for advice, and talking to a mentor (Aulén et al. 2021). Griffith et al. (1999) found that social support at work is significantly negatively associated with teachers’ job stress.

### Summarizing analysis model

The underlying analysis model in this paper shown in Fig. 2 represents a synthesis of the previously described APIM and assumptions on coping approaches, interdependencies in dyadic interactions, and social support. The linkage enables a transfer of this modified model to the context of teacher training, in that on the one hand, the resource of social support and more specifically dyadic support while, on the other hand, the effects of coping strategies can be unified. In the considered context, the dyad consists of a trainee teacher, i.e., a *support recipient* (hereafter also referred to as P_1_) and a supporting person, the *source of support* (hereafter also referred to as P_2_). The coping strategies are considered as the independent behaviors. The impact these may have on the support recipient’s stress is of interest. This study is limited to the partner effect P_2_ → P_1_ and accordingly to the actor effect P_1_.

## Research question and hypotheses

It can be stated that the resource of social support within dyads has so far enjoyed comparatively little attention in studies on teacher training. However, especially because social support constitutes a contextual resource with regard to resilience in teacher training, the inclusion of dyadic supportive relationships appears to be of crucial importance. Accurately, Johnson et al. (2014, p. 532) point out that “an in-depth understanding of the interplay of personal and contextual factors around early career teachers’ experiences” is lacking and that a social conception potentially allows new insights into interpersonal dynamics within and beyond the school context. To our knowledge, no previous study on teacher training refers to dyadic support in combination with corresponding coping strategies. Also, existing studies mostly have included only pre-selected interaction partners and accordingly disregarded other potential sources of support (cf. Richter et al. 2011).

First, the question arises as to which people represent essential sources of social support for trainee teachers. Subsequently, it is of interest how coping efforts in dyadic support interactions affect reported stress during teacher training. In this regard, the effects on stress symptoms experienced by support recipients will be analyzed based on their own coping strategies and, in particular, on those of the sources of support. Based on the literature reviewed, we hypothesize the following:

1. When choosing a source of support, trainee teachers primarily draw on individuals who are/were facing the same stressors or work in the same context (see “Potential sources of support in teacher training” Section).
2. The higher the trainee teacher’s level of coping strategies, with respect to support-seeking, problem-focused, and emotion-regulating coping, the lower the experienced physical and psychological stress symptoms and emotional exhaustion, and the higher the level of well-being (see “Coping strategies on the part of the support recipient and their source of support” Section).
3. The coping behavior of the source of support (i.e., the supporting person) moderates the relationship between the coping behavior of the support recipient (i.e., the trainee teacher) and his or her experienced stress symptoms (see “General modeling of dyadic interactions“ and “Coping strategies on the part of the support recipient and their source of support” Sections).

The last two mentioned assumptions represent the actor effect P_1_ (hypothesis 2) and the partner effect P_2_ → P_1_ (hypothesis 3) (see Fig. 2).

## Method

### Data collection and sample

Data collection was conducted by means of an online-based questionnaire in two steps. First, trainee and fully qualified teachers were canvassed via online teacher forums and e-mail lists to participate in the study. After data cleaning, we could use data from 307 (trainee) teachers (190 female, 117 male). About one-fifth of these respondents were in teacher training at the time, whereas about four-fifths of the sample had already completed their teacher training.^1^ The mean time since these respondents completed their teacher training was *M* = 10.10 years (*SD* = 8.35) and the mean age was *M* = 37.63 years (*SD* = 9.36). A total of 15 out of 18 federal states were represented in the sample: most from Baden-Wuerttemberg (53.42%), followed by North Rhine-Westphalia (11.40%), Hesse (11.40%), Bavaria (7.82%), Rhineland-Palatinate (3.91%), Lower Saxony (3.26%), and 8.79% from other federal states. About half of the sample work at a vocational school, one third at a grammar school, and the remaining fifth of the sample work mainly at a secondary or elementary school.

In the second step, respondents were asked to forward an invitation to participate in the study to a person who supported or had supported them in a special way during teacher training. The assignment of the related dyads was made via a hyperlink, in which the case number of the corresponding (former) trainee teacher was automatically given as a reference (a value stored in the data set). In total, complete data of 49 dyads could thus be recorded in the follow-up survey.

Table 1 contains the sample description of the 49 (trainee) teachers (34 female, 15 male) who represent the *support recipients* in the dyad. On average, they were 33.53 years old (*SD* = 8.06, min. = 25, max. = 57). About one third of them were in teacher training at the time of the study (30.61%). For those teachers who had already been trained, teacher training took place on average 6.74 years ago. Most of them completed this training period in the German state of Baden-Wuerttemberg (83.68%) at a vocational school (65.31%). In the following, we use present tense—in the case of trained teachers, however, these are retrospective views of the already completed teacher training. The reduced sample size is due to the fact that not every (trainee) teacher arranged for the questionnaire to be forwarded to a particularly supportive person, or even if this was the case, the requested source of support did not automatically participate in the survey.

**Table 1:** Sample description of the dyad’s support recipients

Characteristic	n	%	M	SD	min.	max.
Gender						
Female	34	69.87				
Male	15	30.61				
Age	49		33.53	8.06	25	57
Teacher training						
Currently in teacher training	15	30.61				
Teacher training completed	34	69.87				
Teacher training completed since (in years)	34		6.74	6.95	1	26
State in Germany						
Baden-Wuerttemberg	41	83.68				
Hesse	2	4.08				
Bavaria	1	2.04				
Rhineland-Palatinate	1	2.04				
Berlin	2	4.08				
Saxony-Anhalt	1	2.04				
Thuringia	1	2.04				
School type						
Vocational school	32	65.31				
General education secondary school	17	34.69				
Other	2	4.08				

*M* mean, *SD* standard deviation, *min.* minimum; *max.* maximum; School type: due to two multiple answers, the absolute und relative frequency of expression exceeds that of the respondents

Table 2 contains the sample description of the *sources of support* that were assigned to the support recipients. The total of *N* = 49 (28 female, 21 male) who were perceived as particularly supportive were on average 40.67 years old (*SD* = 12.89, min. = 19, max. = 69). Almost 45% (*n* = 22) of them were from the school context and were mentors (*n* = 11, 22.45%), fellow trainees (*n* = 5; 10.20%) or other colleagues (*n* = 6, 12.24%). Approximately 55% (*n* = 27) of the sources of support could be allocated to the private environment sector. A large proportion of them felt that their own partner (*n* = 19, 38.78%) was particularly supportive. A further eight recipients of support perceived people from the family environment as very supportive and therefore named their own mother (*n* = 4, 8.16%) or father (*n* = 2, 4.08%), brother (*n* = 1, 2.04%) or another family member (*n* = 1, 2.04%).

**Table 2:** Sample description of the dyad’s sources of support

Characteristic	n	%	M	SD	min.	max.
Gender						
Female	28	57.14				
Male	21	42.86				
Age	49		40.67	12.89	19	69
Relationship to the trainee teacher						
Mentor, supervising teacher	11	22.45				
Fellow trainee	5	10.20				
Colleague at school	6	12.24				
Partner, spouse	19	38.78				
Mother	4	8.16				
Father	2	4.08				
Brother	1	2.04				
Other family member (e.g., cousin, aunt)	1	2.04				
Sources’ context						
School/work environment	22	44.9				
Private environment	27	55.1				

*M* mean, *SD* standard deviation, *min.* minimum; *max.* maximum

### Measures

Based on the literature, we relied on various measurement instruments that refer to different theoretical constructs of interest in this context. These scales were modified in some instances so that the items addressed the survey context. The operationalizations are presented in Tables 3 and 4, using example items and the number of items per scale. Items were presented to subjects for assessment on a five-point Likert-type scale with response categories ranging from (1) *never* to (5) *very often*. All scales show a moderate to high internal consistency with Cronbach’s alpha as a measure for test-score reliability between 0.72 ≤ *α* ≤ 0.94.

#### Sources of support

Initially, all (trainee) teacher were asked in general terms to what extent they have experienced help and support in problem situations during their teacher training from the relevant people. Here, the level of perceived support from various sources of support in teacher training was in turn assessed by means of the above-mentioned Likert-type scale. Subsequently, the participants were asked to indicate by whom they feel most supported during teacher training. By providing the e-mail address of this source of support, the respective person was invited to participate in the study.

#### Coping strategies of support recipients and sources of support

For analyzing participants’ perceptions of coping strategies when facing stress in teacher training, we used the scales *seeking support*, *problem-focused coping*, and *palliative emotion regulation* described by Lohaus et al. (2018).^2^ These three dimensions were adapted accordingly for the present study and implemented in both questionnaires, with respect to the support recipients as well as to the sources of support. With regard to the latter, the respondents of the partner assessment were asked to imagine a stressful situation in everyday life and to indicate how they generally deal with it.

**Table 3:** Operationalization of coping strategies

Dimension	Items	α	Example item
			When facing stress in teacher training (*support recipients*)/When facing a stressful situation in everyday life (*sources of support*)
Seeking support	5	.85/.86	… I let/had someone help me
Problem-focused coping	6	.78/.86	… I start/started tackling the problem
Palliative emotion regulation	6	.84/.87	… I try/tried to do something to relax

Items adapted from Lohaus et al. (2018); α = Coping P_1_ (support recipients) / Coping P_2_ (sources of support)

#### Stress symptoms among support recipients

Concerning *stress symptoms*, (trainee) teachers were asked how often they experienced physical stress symptoms, psychological symptoms (subscales: anger, sadness, anxiety, well-being) and emotional exhaustion during teacher training. The items of physical and psychological symptomatology were all obtained from the questionnaire of Lohaus et al. (2018). The items of emotional exhaustion were taken from the German version of the Maslach Burnout Inventory by Maslach and Jackson (1981), translated by Barth (1985).

**Table 4:** Operationalization of stress symptoms

Dimension	Items	α	Example item
Physical symptoms	6	.84/.72	During teacher training I have/had headaches
Psychological symptoms			
Anger	4	.87/.83	During teacher training I am/was irritable
Sadness	4	.88/.85	During teacher training I am/was unhappy
Anxiety	4	.87/.86	During teacher training I am/was tense
Well-being	4	.94/.89	During teacher training I am/was cheerful
Emotional exhaustion	4	.86/.80	During teacher training I feel/felt emotionally drained from my work

Items adapted from Barth (1985), Lohaus et al. (2018), and Maslach and Jackson (1981); α = Stress symptoms P_1_ (support recipients) entire sample / Stress symptoms P_1_ (support recipients) dyadic survey

### Data analysis

In addition to the descriptive methods for calculating the scale characteristics of all constructs, Pearson product-moment correlations quantifying the linear relationship between the (sub-)scales were calculated using SPSS 25 software (IBM, Chicago, USA). Furthermore, moderator analyses were conducted with the facets of support recipients’ stress symptoms as dependent variables, support recipients’ coping strategies as predictors, and the coping strategies of the source of support as moderator variables. As literature shows, analysis of dyadic data can be conducted with relatively small sample sizes for actor and partner data (e.g., Tambling et al. 2011). Kenny et al. (2006) recommend a minimum of 25 dyads for conducting dyadic data analysis. In our dyadic data set, we had data from 49 dyads, and we used bootstrap regression models with bias-corrected and accelerated confidence intervals; results are based on 1000 bootstrap samples (cf. Nikitina et al. 2019). Interpreting the magnitude of effect sizes, we refer to Cohen (1992).

In the first step of our analysis, the sample of *N* = 307 was used to determine which groups of people were generally perceived as highly supportive and which explicit source of support was named as a particularly supportive interaction partner (“Perceived support from different sources of support” Section). In the second step, the dyadic coping effects described above were examined on the basis of the reduced sample of *N* = 49 (“Stress management and stress experience in the context of dyadic support” Section).

## Findings

### Perceived support from different sources of support

Table 5 presents the descriptive findings on the generally perceived support provided by different sources of support. These findings are based on the entire sample of (trainee) teachers.

**Table 5:** Perceived support from diverse sources of support (entire sample)

	M	SD	Never	Rarely	Sometimes	Often	Very often
Professional context							
Fellow trainees	4.14	.92	5 (1.80%)	11 (3.96%)	38 (13.67%)	111 (39.93%)	113 (40.65%)
Colleagues at school	3.88	.90	2 (.72%)	21 (7.55%)	57 (20.50%)	127 (45.68%)	71 (25.54%)
Mentors^a^	3.73	1.09	12 (4.32%)	29 (10.43%)	54 (19.42%)	110 (39.57%)	73 (26.26%)
Seminar teachers^b^	3.01	1.07	26 (9.42%)	58 (21.01%)	100 (36.23%)	71 (25.72%)	21 (7.61%)
School principal	2.56	1.20	66 (23.83%)	71 (25.63%)	78 (28.16%)	44 (15.88%)	18 (6.50%)
Private context							
Partner, spouse	3.62	1.41	37 (13.75%)	28 (10.41%)	31 (11.52%)	78 (29.00%)	95 (35.32%)
Friends	3.03	1.29	48 (17.52%)	43 (15.69%)	73 (26.64%)	74 (27.01%)	36 (13.14%)
Parents	2.80	1.49	80 (29.20%)	45 (16.42%)	48 (17.52%)	51 (18.61%)	50 (18.25%)
Other family members	2.10	1.27	135 (49.45%)	38 (13.92%)	46 (16.85%)	45 (16.48%)	9 (3.30%)

*M* mean, *SD* standard deviation; 269 ≤ *n* ≤ 278; *n* (in %), the reduced number of persons results from the fact that not all of the 307 respondents provided information on the perceived support of various sources of support; ^a^supervising teachers at the schools where the trainee teachers are employed; ^b^supervising teachers for specific subjects at the colleges of didactics and teacher education

It is striking that on average, with *M* = 4.14, fellow trainees are perceived as most supportive by far. Only five respondents (1.80%) *never* receive assistance from this group of people. In contrast, 113 (40.65%) *very often* receive support from fellow trainee teachers. In addition, colleagues at the school (*M* = 3.88) and the mentor (*M* = 3.73) are *often* perceived as supportive. In the private environment, one’s own partner or spouse is perceived as most supportive (*M* = 3.62). More than a third (35.32%) of the participants (*n* = 95) confirm that support *very often* comes from one’s own partner or spouse. The two potential sources of support that on average provide the least support are the school principal (*M* = 2.56) and relatives (*M* = 2.10). Altogether, and with a frequency of *f* = 296, participants indicated that they *very often* (or *often: f* = 463) receive support from people who belong to the school or training context, i.e., mentors or supervising teachers, fellow trainees, colleagues, seminar teachers or school principals. In contrast, roughly the same number of people stated that people from the private setting *never* (*f* = 300) provide supportive assistance. The overall mean difference between sources from work environment (*M* = 3.46, *SD* = 0.69) and sources from private environment (*M* = 2.90, *SD* = 1.02) is significant at *p* < 0.001.

Concerning the entire sample, correlations between (trainee) teachers’ stress symptoms and perceived support show the following: perceived support from *sources from the work environment* is significantly negatively associated with anger (*r* = –0.23, *p* = 0.001), sadness (*r* = –0.32, *p* < 0.001), anxiety (*r* = –0.18, *p* = 0.005) and emotional exhaustion (*r* = –0.14, *p* = 0.038), and it is significantly positively associated with well-being (*r* = 0.34, *p* < 0.001). In contrast, perceived support from *sources from the private environment* is significantly positively correlated just with anger (*r* = 0.14, *p* = 0.038).

With regard to the one reference person perceived as most supportive during teacher training, mentors and supervising teachers were named most frequently, followed by partners, fellow trainees, colleagues at school, parents, good friends, seminar teachers, siblings, the school principal, and other family members (e.g., cousin, aunt) (Table 6). Altogether, however, 164 indicated a person associated with the training context as a particularly supportive reference contact. In total, 99 sources of support can be allocated to the private environment. The chi squared test indicates that the difference in frequencies is significant at *p* < 0.001 (the calculation was made without the information on “Other (unspecified), n = 4”).

**Table 6:** Reference persons perceived as particularly supportive during teacher training (entire sample)

Reference persons	F	%
Mentor^a^	66	24.72
Partner, spouse	61	22.84
Fellow trainee	60	22.47
Colleague at school	30	11.23
Parent	20	7.50
Good friend	11	4.12
Seminar teacher^b^	6	2.25
Sibling	5	1.87
Other (unspecified)	4	1.50
School principal	2	.75
Other family member (e. g., cousin, aunt)	2	.75

*F* frequency; the reduced number of persons results from the fact that not all of the 307 respondents provided information on the perceived support of various sources of support; ^a^supervising teacher at the schools where the trainee teachers are employed; ^b^supervising teacher for specific subjects at the colleges of didactics and teacher education

### Stress management and stress experience in the context of dyadic support

#### Descriptive results and correlations

Table 7 contains descriptive data and Pearson correlation coefficients of the dyadic survey.

**Table 7:** Descriptive results and correlations of the dyadic survey (*N* = 49)

		*M*	*SD*	*α*	(1)	(2)	(3)	(4)	(5)	(6)	(7)	(8)	(9)	(10)	(11)	(12)	(13)	(14)	(15)	(16)	(17)	(18)
(I) Coping P_1_	(1) Seeking support P_1_	3.58	.81	.85																		
	(2) Problem-focused coping P_1_	4.07	.49	.78	.19																	
	(3) Palliative emotion regulation P_1_	2.50	.77	.84	− .05	− .05																
(II) Stress symptoms P_1_	(4) Physical symptoms P1	2.04	.63	.72	.48^**^	.07	.08															
	(5) Anger P_1_	2.68	.76	.83	.33^*^	.26	− .15	.47^**^														
	(6) Sadness P_1_	2.47	.77	.85	.32^*^	.19	− .07	.54^**^	.64^**^													
	(7) Anxiety P_1_	3.53	.84	.86	.29^*^	− .10	− .04	.60^**^	.59^**^	.68^**^												
	(8) Well-being P_1_	3.55	.63	.89	− .05	.12	.36^*^	− .23	− .38^**^	− .47^**^	− .49^**^											
	(9) Emotional exhaustion P_1_	3.38	.84	.80	.34^*^	.12	− .12	.53^**^	.61^**^	.54^**^	.72^**^	− .50^**^										
(III) Sociodemographics P_1_	(10) Gender P_1_	69.87% female			− .53^**^	− .26	− .09	− .26	− .02	− .23	− .10	− .08	− .11									
	(11) Age P_1_	33.53	8.06		− .09	.16	− .03	− .08	.01	− .05	− .23	− .07	− .14	.11								
	(12) Currently in teacher training^a^ P_1_	30.61%			− .15	.02	.10	− .09	− .21	.15	− .06	.09	− .33^*^	.06	.43^**^							
	(13) Teacher training completed for (years)^b^ P_1_	6.74	6.95		.04	.13	− .06	.07	.16	− .08	− .14	− .06	.06	.06	.95^**^	−						
(IV) Coping P_2_	(14) Seeking support P_2_	3.27	.79	.86	.03	.03	− .02	.01	.14	.12	− .05	.02	.09	.15	.13	− .17	.31					
	(15) Problem-focused coping P_2_	4.03	.58	.86	− .04	− .15	− .24	.08	.10	.13	.09	− .15	− .06	.36^*^	.12	.05	.14	.26				
	(16) Palliative emotion regulation P_2_	2.68	.70	.87	.05	.14	.26	.18	.17	.14	− .07	.16	− .01	− .05	− .16	− .05	− .12	− .03	− .07			
(V) Sociodemographics P_2_	(17) Gender P_2_	57.14% female			.08	− .12	.00	− .05	− .18	− .15	− .03	.12	− .18	− .04	− .12	− .05	− .14	− .57^**^	.10	.05		
	(18) Age P_2_	40.67	12.89		.15	.14	− .18	.25	.01	.10	− .01	− .30^*^	.00	− .19	.43^**^	.16	.52^**^	− .14	.03	− .08	.04	
	(19) Sources’ context^c^	44.9% from the work environment			.14	.04	.22	.11	− .05	− .09	.07	.25	.15	-.11	− .44^**^	− .42^**^	− .31	− .14	.01	− .16	.28^*^	− .20

*P*_*1*_ support recipient, *P*_*2*_ source of support; **p ≤ .01, *p ≤ .05; *α* = Cronbach’s alpha; Coping and stress symptoms: five-point Likert-type scale (1 = never, 2 = rarely, 3 = sometimes, 4 = often, 5 = very often); Gender: (0 = female, 1 = male); ^a^Currently in teacher training: (0 = yes, 1 = no); ^b^Teacher training completed: number of years passed since completion of teacher training; ^c^Sources’ context (0 = school/work environment, 1 = private environment)

With regard to coping with stress, it is noticeable that the *support recipients* on average often chose a problem-focused approach (*M* = 4.07, *SD* = 0.49) and they indicated a support-seeking behavior on average *sometimes* to *often* (*M* = 3.58, *SD* = 0.81). Respondents chose an emotion-oriented coping approach on average *rarely* to *sometimes* (*M* = 2.50, *SD* = 0.77). With respect to stress, (trainee) teachers stated that on average they rather *rarely* experienced physical symptoms (*M* = 2.04, *SD* = 0.63). In contrast, psychological stress symptoms occurred more often, but nevertheless only *rarely* to *sometimes*. In this regard, a distinction is made between anger, sadness, and anxiety. The latter shows the strongest level on average (*M* = 3.53, *SD* = 0.84), followed by anger (*M* = 2.68, *SD* = 0.76) and sadness (*M* = 2.47, *SD* = 0.77). Respondents experienced well-being *sometimes* to *often* with a mean of *M* = 3.55 (*SD* = 0.63). The level of emotional exhaustion is slightly above the scale mean (*M* = 3.38, *SD* = 0.84).

The coping strategy of seeking support shows significant *positive* correlations to all physical and psychological stress symptoms. Physical symptomatology indicates the highest effect size with *r* = 0.48. All other effect sizes rank between 0.29 ≤ *r* ≤ 0.34. Additionally, it should be mentioned that palliative emotion regulation is significantly positively associated to psychological well-being (*r* = 0.36). There is a significant negative correlation between gender and support-seeking coping (*r* = − 0.53). Consequently, female trainee teachers seek more support from others. In addition, trainee teachers are significantly more inclined to report emotional exhaustion during their teacher training than are fully qualified teachers in retrospect (*r* = − 0.33).

The coping behavior of the *sources of support* shows comparable scale values to the coping of the support recipient. On average, the respondents also *often* use a problem-focused coping approach (*M* = 4.03, *SD* = 0.58). Support-seeking coping has a mean of *M* = 3.27 (*SD* = 0.79), followed by palliative emotion regulation, which is the least prominent scale (*M* = 2.68, *SD* = 0.70).

The sociodemographic data of the sources of support show only a few significant correlations to all other scales. Once again, a significant negative correlation is found between gender and the coping strategy seeking support (*r* = − 0.57). The coping behavior seeking support is thus practiced more often by women. The age of both interaction partners correlates positively with a moderate effect size (*r* = 0.43).

#### Results of the moderator analysis

In the course of determining the *partner effects*, moderator analyses were calculated in the dyadic study. Analyses are based on the possible combinations between the coping behavior of the support recipient (seeking support P_1_, problem-focused coping P_1_, palliative emotion regulation P_1_) and his or her stress symptoms (physical symptoms, anger, sadness, anxiety, well-being, emotional exhaustion). In this regard, the potential moderating coping behavior of the source of support (seeking support P_2_, problem-focused coping P_2_, palliative emotion regulation P_2_) is of interest. With respect to the relationship just described, this yields a total of 54 possible combinations of variables. With reference to Table 7, we controlled for the variables *age P*_*2*_ in respect to *well-being* as well as *currently in teacher training P*_*1*_ in view of *emotional exhaustion*. For reasons of clarity, only the models with significant interaction effects are reported and described in more detail below.

With regard to the relationship between P_1_’s palliative emotion regulation and this person’s stress, we found three combinations in which P_2_’s coping behavior acts as a moderator. Anger is *intensified*, while well-being is *decreased* (see Table 8 models a, b, and c). In the first interaction term (model a), P_2_’s problem-focused coping moderates the relationship between P_1_’s palliative emotion regulation and this person’s experience of anger (*β* = 0.332, *p* = 0.015). Similarly, the coping strategy seeking support of P_2_ acts as a moderator in regard to the just-mentioned association concerning the feeling of anger (*β* = 0.278, *p* = 0.057). Yet, one should note that is true only for a significance level of 10%. Additionally, P_1_’s experience of well-being shows a decline through the significant moderator effect of precisely that support-seeking coping approach (*β* = − 0.288, *p* = 0.046). With respect to the relationship between support-seeking coping and emotional exhaustion of P_1_, the problem-focused coping behavior of the source of support (Table 8 model d) acts as a buffer in this regard (*β* =  − 0.253, *p* = 0.034). The corrected R^2^ of all reported models and the effect sizes obtained from them can also be found in Table 8.

**Table 8:** Interaction effects of the moderator analyses (*N* = 49)

						BCa 95% CI	
Models	B	Bias	SE(B)	*β*	*p*	LB	UB
*Model a*: d.v. *Anger P*_*1*_							
(Constant)	2.749	0.004	0.113		0.001	2.529	2.980
Palliative emotion regulation P_1_	− 0.156	− 0.005	0.164	− 0.160	0.332	− 0.463	0.144
Problem-focused coping P_2_	0.049	− 0.009	0.185	0.037	0.786	− 0.350	0.389
Interaction PER P_1_ × PC P_2_	0.597	− 0.006	0.258	0.332	0.015	0.077	1.075
*Model b*: d.v. *Anger P*_*1*_							
(Constant)	2.692	0.007	0.102		0.001	2.454	2.922
Palliative emotion regulation P_1_	− 0.114	− 0.013	0.140	− 0.117	0.387	− 0.389	0.119
Seeking support P_2_	0.162	− 0.005	0.150	0.167	0.281	− 0.120	0.441
Interaction PER P_1_ × SS P_2_	0.332	− 0.014	0.181	0.278	0.057	− 0.059	0.644
*Model c*: d.v. *Well-being P*_*1*_							
(Constant)	3.984	− 0.020	0.298		0.001	3.461	4.466
Palliative emotion regulation P_1_	0.240	0.011	0.108	0.294	0.039	0.016	0.481
Seeking support P_2_	− 0.038	0.001	0.116	− 0.047	0.752	− 0.265	0.204
Interaction PER P_1_ × SS P_2_	− 0.288	− 0.004	0.151	− 0.288	0.046	− 0.548	− 0.002
Age P_2_	− 0.011	0.000	0.007	− 0.224	0.103	− 0.026	0.002
*Model d*: d.v. *Emot. exhaust. P*_*1*_							
(Constant)	3.683	− 0.014	0.236		0.001	3.183	4.070
Seeking support P_1_	0.240	− 0.005	0.147	0.234	0.102	− 0.016	0.522
Problem-focused coping P_2_	− 0.208	0.010	0.211	− 0.142	0.287	− 0.636	0.272
Interaction SS P_1_ × PC P_2_	− 0.302	− 0.021	0.184	− 0.253	0.034	− 0.663	− 0.044
Curr. teacher training P_1_	− 0.455	0.010	0.272	− 0.253	0.092	− 1.019	0.128

*d.v.* dependent variable, *PER* Palliative emotion regulation, *PC* Problem-focused coping, *SS* Seeking support, Curr. teacher training P_1_ (Currently in teacher training, 0 = yes, 1 = no), *BCa* Bias-corrected and accelerated, *LB* lower bound, *UB* upper bound

*Model a*: R-square = 0.135, adjusted R-square = 0.076, SE of the Estimate = 0.726

*Model b*: R-square = 0.115, adjusted R-square = 0.054, SE of the Estimate = 0.735

*Model c*: R-square = 0.269, adjusted R-square = 0.201, SE of the Estimate = 0.564

*Model d*: R-square = 0.243, adjusted R-square = 0.173, SE of the Estimate = 0.765

**Fig. 3:**
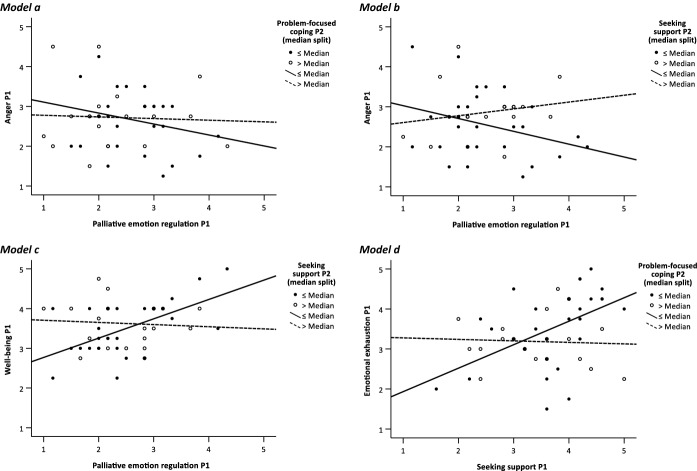
Illustration of the interaction effects

All significant interaction effects found are visualized in Fig. 3. Median splits regarding problem-focused coping of P_2_ (Fig. 3 models a and d) and the coping strategy seeking support of P_2_ (Fig. 3 models b and c) are used to illustrate the linear group-specific growth lines.

## Discussion

### Summary of findings

This paper attempts to contribute to the analysis of contextual resources that may promote teacher resilience by identifying sources of support in teacher training as well as investigating interdependencies in dyadic coping interactions. First, we examined which people represent sources of social support in teacher training and who is perceived as a particularly supportive interaction partner. Following Thoits (1986) and McPherson et al. (2001), among others, it was assumed (*hypothesis 1*) that, when choosing a source of support, people will rely primarily on socially similarly situated persons and that the resulting support will be perceived as effective and helpful. The descriptive results presented confirm the assumption. In problem situations in teacher training, support recipients most frequently perceive supportive assistance from fellow trainees, colleagues, and mentors. These findings are in line with Mansfield et al. (2016), who found that especially teacher-leader and teacher-teacher relationships were frequently mentioned contextual resources influencing resilience. Tables 5 and 6 show that, overall, sources of support originating from the school context are very often perceived as supportive and a majority of (trainee) teachers chose a school-context-related person as their most supportive reference and interaction partner. The results suggest that these individuals are or have been exposed to comparable stressors. Support services from this group of individuals are particularly effective (cf. Thoits 1986). Indeed, a study on help-seeking behavior of student teachers found that prospective teachers most frequently sought the help of peers when confronted with challenges, followed by approaching mentoring teachers (Hsu 2005). Moreover, Le Cornu (2013, p. 6) describes peers of early career teachers as having a “key role” when it comes to being resilient over a longer period of time. In addition, there is also a statistically significant positive correlation between the age of both coping partners, which means that supportive dyads are predominantly composed of interaction partners of similar age. Concerning the relationship levels, support recipients nevertheless resort to people from the school context as well as to privately known individuals. This result is in accordance with Papatraianou and Le Cornu (2014), who argue that there is no need for a distinction between two different groups of people regarding the provision of support. It rather has to be acknowledged that not only formal processes of support but also informal supportive interactions offered by professionals as well as private network members are fundamental to enhancing early career teacher resilience.

*Hypothesis 2* is concerned with the actor effect, i.e., the effect of the coping behavior of the support recipient on his or her stress symptoms. It was assumed that the higher the level of coping strategies, the lower the experience of stress. This is true for well-being. A high level of palliative emotion regulation is associated with higher well-being. This is plausible, as an inward-oriented approach involving relaxing interventions calms the mind. In contrast, it is striking that physical and psychological stress symptoms are significantly positively correlated with the coping strategy seeking support. Thus, although this result is not consistent with the hypothesis, the present findings do not contradict the key messages of this study, but rather highlight the importance of social support. The direction of effect of the correlation must only be interpreted differently here. No statements can be made about causality, which is why it is entirely possible that a need for help and assistance is caused by stress symptoms. Thus, if the trainee teacher is not well both physically and psychologically, he or she will seek support.

In the course of addressing the partner effect and thus *hypothesis 3*, the moderation effects come into focus (Table 8, Fig. 3), as a better understanding of the “dynamic and complex interplay among individual, relational, and contextual conditions that operated over time to promote teacher resilience” is needed (Johnson et al. 2014, p. 534). It was assumed that the coping behavior of the source of support moderates the relation between the coping behavior of the support recipient and his or her stress symptoms. This is true in four possible combinations, so that we can partially confirm the assumption. Yet it must be stated these results show merely first indications of possible interdependencies in dyadic coping interactions during teacher training. It becomes apparent that support recipients who particularly engage in palliative emotion regulation and at the same time have a source of support that predominantly shows a proactive attitude or an outward going approach, experience a higher stress response. The source of support’s problem-focused coping intensifies the experience of anger with support recipients. Likewise, the coping strategy of seeking support on the part of the source of support has a stress-reinforcing effect. Anger is more pronounced in these individuals and it also results in a reduced well-being. In all three cases, there is a mismatch between the support recipients needs and the type of support provided. This implies that relationships with coping partners may not only serve as protective factors but that certain interactions are more likely to constitute risk factors for resilience. The reviews of Beltman et al. (2011) and Beltman (2021) also underline contextual challenges occurring through relationships. However, these reviews and the included studies tend to focus for example on obstructive relationships with colleagues or on work environments that are non-conducive, such as unsupportive leadership staff or mentors. Referring back to our findings, it can be concluded that it is counterproductive if support recipients who resort to calming activities in stressful situations and demonstrate an inward-oriented way of dealing with stress and the resulting emotions rely on sources of support that favor a more outward-oriented or problem-addressing approach. In principle, these approaches in fact differ fundamentally, as palliative emotion regulation is more about personal withdrawal as well as gaining distance from the stressor. Yet seeking support is expressed in mobilizing others and problem-focused coping is about confronting the predicament. The literature shows that emotion-focused coping is described as less adaptive compared to problem-focused coping (e.g., Aulén et al. 2021; Pogere et al. 2019). According to our findings, an individual perspective of labeling a coping strategy as “adaptive” or “maladaptive” possibly falls short. Thus, an individual’s coping strategies have to be interpreted in the context of the individual’s social relationships and subsequent stress symptoms may not only be a result of the individual's coping efforts, but also of the interaction between the individual and his or her social context. Although existing findings point to the relevance of person-environment congruence for person outcomes (e.g., Eagan and Walsh 1995), little is yet known about such interdependencies in the context of individual coping efforts. Therefore, it is important that future studies focus more on person-environment congruence in the context of stress coping and resilience in teaching and teacher training.

The final interaction effect found is reflected in a reduction of emotional exhaustion insofar as P_1_ follows a support-seeking strategy and P_2_ shows problem-focused coping (Table 8, Fig. 3). Thus, it appears that the two coping strategies interact constructively with respect to the feeling of emotional exhaustion. This makes sense because, if one turns to someone for support and that person responds by directly trying to tackle and solve the problem, it eases the person’s burden. The focus lies on the attempt to directly change the emotional impact that a stressor triggers and thereby minimize emotional exhaustion. This finding possibly reflects the importance of supportive relationships on early career teachers’ resilience (Johnson et al. 2014). The crucial role of collective coping, social embeddedness and familiar social surroundings has also been reemphasized in the COVID-19 pandemic. In fact, social distancing was among the most discomforting aspects of distance teaching and led to a deterioration in professional well-being. Multiple factors that maintained teachers’ well-being included for example contextual work-related aspects, such as collegial support and cooperation, resilience, and coping strategies (Hascher et al. 2021).

In consideration of all reported moderation effects, it can be concluded that the fit of two interaction partners determines the success of dyadic coping interactions. In a similar vein, Frisch et al. (2014) argue that a match of underlying identities increases the effectiveness of social support. Specifically, their experimental evidence indicates that social support is more likely to buffer stress responses when there is a shared social identity between the support recipient and the source of support.

### Limitations of the study and implications for further research

Although we provide initial insight into coping interdependencies and contextual factors influencing resilience in teacher training within the framework of social support—a topic that, to our knowledge, has not been investigated in the present form—limitations of the approach should be mentioned. First of all, it cannot be ruled out that the sample might be selective as the survey was conducted on a voluntary basis. It is possible that only individuals who are predominantly well-integrated in a professional and/or personal network participated in this study. Secondly, the dyadic analysis is composed of a reduced sample size. The smaller sample size is most likely due to the fact that the possibility of participation of a source of support was initiated only by an active invitation of a support recipient. The voluntary provision of the e-mail address is thus decisive. With regard to sharing e-mail addresses, many people may be rather reluctant. In addition, and above all, the selected person must be willing to participate in the survey. Furthermore, it should be noted that not only trainee teachers participated in the survey, but also fully trained teachers who had completed their teacher training several years ago. However, the number of years that had passed since the completion of teacher training was not significantly correlated with any of the stress and coping variables (Table 7). Furthermore, training status (currently in teacher training or teacher training already completed) was significantly correlated only with emotional exhaustion.

Moreover, the direction of action was assumed by the evaluation procedures of multiple regression or moderator analyses and by the designed model, however, causality cannot be assumed in a cross-sectional study. In this regard, Cook and Kenny (2005) note that longitudinal studies are not necessarily needed to test the APIM, as effects can also be defined more generally as the effects of a characteristic or behavior in resulting outcomes. Nevertheless, and in order to account for the dynamic perspective of dyadic support interdependencies in teacher training, a longitudinal design in following studies would be worthwhile. Besides, coping strategies were seen as rather rigid and exclusive, as individuals do not solely follow one approach, but rather apply a dynamically varied mix of coping strategies and their application. Further research should consider coping resources in teaching and teacher training in a broader sense that take not only the individual perspective into account but also the dyads and social networks (trainee) teachers are integrated in. In consideration of that, for instance, empathy, sympathy, shared social identity, and other relational characteristics play a crucial role in the selection of which person one approaches to seek support (Frisch et al. 2014; McPherson et al. 2001; Verbrugge 1977), further research should also focus on characteristics of the relationships between support recipients and their sources of support. In the context of teacher training, Kärner et al. (2021b) found, for example, that the more transparent, fairer, and more trusting and less ambivalent the relationship with their mentors was, the milder were the stress symptoms of the support recipients. Consequently, social competence seems to be essential for building resilience in teacher training (cf. Mansfield et al. 2016).

As stress and coping processes play crucial roles in teaching and teacher training, such processes must be considered within a system of complex social interdependencies. Thus, succeeding or failing in teacher training may not only be a product of the individual but rather one of the private and work-related networks an individual is integrated in. As illustrated by discussions about relational resilience, the role of relationships in the resilience process is of crucial importance (Gu 2014; Le Cornu 2013). Professional learning opportunities for supervising teachers as well as trainee teachers in how to establish and develop supportive environments and collegial social networks to interact effectively might serve as beneficial factors promoting resilience through contextual resources in teacher education (cf. Mansfield et al. 2016; Papatraianou and Le Cornu 2014). Accordingly, “[p]re-service teachers should develop a strong conceptual understanding of resilience, specifically its dynamic and multifaceted nature. This means not only appreciating personal strengths and limitations, but developing awareness of the contextual resources and coping strategies that can promote resilience” (Mansfield et al. 2016, p. 21). Le Cornu (2013) also puts emphasis on strengthening relationships rather than merely working on individual strengths and weaknesses to increase trainee teachers’ capacity to be resilient. Finally, teacher training programs should place a primary focus on individual and especially contextual protective factors by providing opportunities for collective and supportive coping.

## Data Availability

The data will not be shared publicly because for data protection reasons.

## Appendix

See Table 9.

**Table 9:** Items for assessing coping strategies of support recipients and sources of support

Dimension	Items
Seeking support	… I tell/told someone what happened
	… I let/had someone help me
	… I let/had myself be comforted by someone
	… I tell/told someone how I felt about it
	… I ask/asked someone to help me with the problem
Problem-focused coping	… I try/tried to do better next time
	… I start/started tackling the problem
	… I choose/chose a way to solve the problem
	… I think/thought about how to solve the problem
	… I try/tried hard so it does/did not happen again
	… I change/changed something so that things run better
Palliative emotion regulation	… I rest/rested
	… I recover/recovered to gather new strength
	… I treat/treated myself to a break for the time being
	… I make/made myself comfortable for the time being
	… I try/tried to do something to relax
	… I do/did something I can/could really enjoy

Items adapted from Lohaus et al. (2018); Introductory text: “When facing stress in teacher training (support recipients) .../When facing a stressful situation in everyday life (sources of support) ...”

## Abbreviations

APIM: Actor-Partner Interdependence Model
P_1_: Partner 1 (support recipient)
P_2_: Partner 2 (source of support)

## Footnotes

1 Fully trained teachers answered all training-related questions by recollection.

2 Although the questionnaire was developed by Lohaus et al. (2018) to assess stress and stress management in childhood and adolescence, many items are formulated in such a general way that they can also be applied to adults. If this was not the case for an item, we adapted it accordingly to the context of adults. The items for assessing coping strategies of support recipients and sources of support can be found in the appendix (Table 9).
